# Infrared nanoimaging of neuronal ultrastructure and nanoparticle interaction with cells

**DOI:** 10.1039/d3nr04948e

**Published:** 2024-03-05

**Authors:** George E. Greaves, Leanne Allison, Pedro Machado, Corinne Morfill, Roland A. Fleck, Alexandra E. Porter, Chris C. Phillips

**Affiliations:** a Experimental Solid State Group, Department of Physics, Imperial College London SW7 2BW UK george.greaves15@imperial.ac.uk; b Department of Materials and London Centre for Nanotechnology, Imperial College London SW7 2AZ UK; c Centre for Ultrastructural Imaging, Kings College London SE1 1UL UK; d Randall Centre for Cell and Molecular Biophysics, Kings College London SE1 1YR UK

## Abstract

Here we introduce scattering-type scanning near-field optical microscopy (s-SNOM) as a novel tool for nanoscale chemical-imaging of sub-cellular organelles, nanomaterials and of the interactions between them. Our setup uses a tuneable mid-infrared laser and a sharp scanning probe to image at a resolution substantially surpassing the diffraction limit. The laser can be tuned to excite vibrational modes of functional groups in biomolecules, (*e.g.* amide moieties), in a way that enables direct chemical mapping without the need for labelling. We, for the first time, chemically image neuronal ultrastructure, identify neuronal organelles and sub-organelle structures as small as 10 nm and validate our findings using transmission electron microscopy (TEM). We produce chemical and morphological maps of neurons treated with gold nanospheres and characterize nanoparticle size and intracellular location, and their interaction with the plasma membrane. Our results show that the label-free nature of s-SNOM means it has a ‘true’ chemical resolution of up to 20 nm which can be further improved. We argue that it offers significant potential in nanomedicine for nanoscale chemical imaging of cell ultrastructure and the subcellular distribution of nanomaterials within tissues.

## Abstract 2

We use infrared nanoscopy to image subcellular components of hippocampal neurons in a chemistry specific way and to study their interaction with therapeutic nanoparticles.

## Graphical abstract

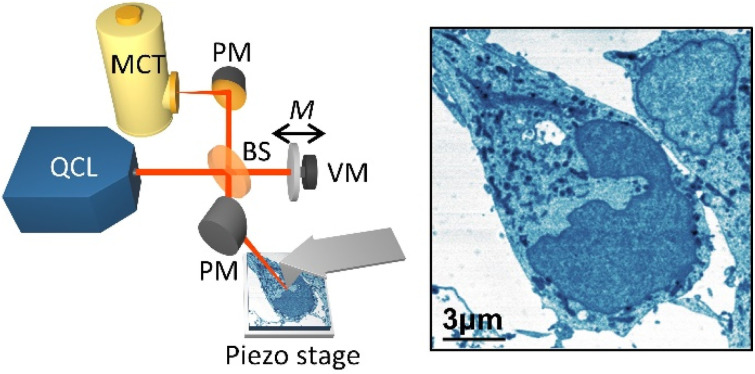

## Introduction

Nanoscale imaging of cell ultrastructure, molecular species and chemical compounds in tissues is key for understanding cell function and pathology.^1^ It is also paramount in nanomedicine due to the recent development of theranostics based on nanoparticles (NP),^2–4^ with examples that include non-metal materials such as lipids and silica, or noble metals such as gold (AuNP).^5,6^ These nano-therapeutics can be engineered to be small enough to cross cellular barriers whilst retaining a high surface area that can carry large cargos. They can perform multiple functions including cell targeting, bioimaging and drug delivery.^7–9^ A key challenge, however, is to characterize the cellular uptake of nanomedicines, their chemical transformations and intracellular locations, and the changes they make (induced by processes such as oxidation) to the biochemistry of organelles.

Fluorescence microscopies, including recent super-resolution techniques^10–12^ and single-molecule localization microscopy (SMLM),^13,14^ have achieved spatial resolutions down to ∼10 nm,^13^ and have been used for cellular imaging and for detecting fluorescently-labelled nanomaterials inside cells.^15^ However, these methods are limited^13^ in that they visualize only the position of the fluorescent label, *i.e.* not the target molecules themselves.^16^ Often they perturb the biology of samples^13^ and the labels need to be selected *a priori*; with the consequence that unforeseen but important molecules may be missed due to the lack of appropriate labels.

Electron microscopy (EM) offers higher spatial resolution than fluorescence microscopy and, when combined with spectroscopy techniques such as electron energy loss spectroscopy (EELS) and energy dispersive X-ray spectroscopy (EDX), can generate information about the elemental composition of nanomaterials. It has been applied widely to image the structure of cells and the interaction of nanomaterials with cell organelles.^17^ Developments in cryo-electron microscopy (EM), involving rapidly vitrifying cells and imaging them at cryogenic temperatures, can also be used to improve structural and chemical preservation of cells and biomolecules by preserving the cells in their near-native state.^18^ Although EM imaging of the distribution and composition of nanomaterials inside cells is achievable, there is a problem with sample damage.^19^

Optical diffraction tomography can be used to measure the refractive index of biological specimens through quantitative phase imaging.^20,21^ This allows specimens to be identified in a label-free way. However, it is challenging to spatially resolve beyond the diffraction limit and thus to resolve many sub-cellular features.^20^

Here we use scattering-type scanning near-field optical microscopy (s-SNOM); it uses an atomic force microscope (AFM) in conjunction with tuneable infrared (IR) lasers to enable optical imaging that surpasses the diffraction limit.^22^ A laser illuminates the sharp AFM tip which is vibrating close to the sample surface. The backscattered laser light can be analysed to infer the nature of the sample's optical response (both absorption and refractive index) in a very small patch beneath the tip.^23^

Tuning the laser (in our case an array of tuneable IR quantum cascade lasers (QCLs)), enables narrowband imaging across the mid-IR range. It combines nanoscale spatial resolving power with point spectroscopy in the mid-IR, thereby yielding nanoscale chemical sensitivity. Importantly, since the incident field is largely confined to the probe apex, the spatial resolution of s-SNOM is independent of the wavelength. It is instead determined by the tip diameter (5–50 nm)^24^ and can beat diffraction by several decades in the mid-infrared region.

In contrast to TEM, s-SNOM has the advantage of being able to provide cell ultrastructure morphology whilst at the same time inferring chemical species. Such ultrastructural features include cell regions and organelles with enriched species, such as proteins^25–30^ and nucleobases^29^.

s-SNOM and electron microscopy used in tandem can provide complementary information about both the biochemistry and structure of cell organelles and the intracellular destination of nanomaterials. Here, we use multi-wavelength s-SNOM for nanoscale imaging of neurons based on their chemical contrast. We identify components of the cell ultrastructure as small as ∼10 nm and we use biochemical signatures to identify macromolecular components of the cell organelles.

As a direct demonstration of the utility of this approach, we use it to study the interactions between gold nanoparticles (AuNP) and neurons. AuNP are being extensively tested for therapeutic applications and are currently used for bioimaging, for brain tumour theranostics^31,32^ and as probes for correlative light-electron microscopy.^33^

Previously we have engineered peptide-conjugated AuNP (∼40 nm diameter) that were found to protect neurons in models of neurodegenerative diseases, and we studied their interaction with neurons using EM.^34,35^ Here we determine the s-SNOM resolving capability and the optimal imaging modalities for these nanostructures, and we compare the AuNP distribution in the samples imaged by s-SNOM with those in TEM. We show that s-SNOM gives reliable imaging of AuNP within cells whilst mapping the neuronal ultrastructure in a chemistry-sensitive way that can be correlated with structural features from cells from the same culture imaged by TEM. Our results suggest a promising potential for s-SNOM in nanomedicine for structural imaging and for chemical identification of organelles, as well as for locating intracellular nanomaterials.

## Experimental setup

**Fig. 1:**
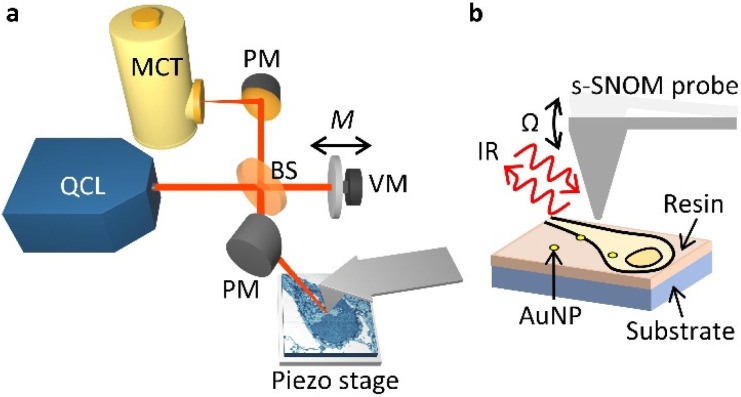
s-SNOM imaging setup. (a) Quantum cascade laser (QCL) light is focused, *via* a beam splitter (BS) and a parabolic mirror (PM), onto a sharp tip oscillating vertically at frequency *Ω*. A reference arm in an interferometric detection scheme is phase modulated at frequency *M* by a vibrating mirror (VM). The backscattered light from the probe is recombined with the reference arm and is detected with a mercury cadmium telluride detector (MCT). (b) Geometry of the tip–sample interaction, scanning ∼70 nm thick sections of resin-embedded neurons treated with gold nanoparticles (AuNP) mounted on silicon substrates.

In our s-SNOM setup (Fig. 1, see Methods for details), a parabolic mirror (PM) focuses mid-infrared laser light onto the tip of a vibrating AFM probe, and establishes a localised electric field at its apex. This electric field is modified by the sample such that the tip-backscattered light (specifically its phase, φ) corresponds to the absorption measured with infrared absorption spectroscopy techniques such as Fourier transform infrared (FTIR) spectroscopy.^36^ This allows one to infer the local optical absorption coefficient of the sample. As the probe is rastered across the sample to build up an image, its vertical position is continuously adjusted to the sample contours so as to maintain constant tapping amplitude (AFM tapping mode).

The optical signal is demodulated at harmonics of the tapping frequency of the probe, *Ω*, split into sidebands separated by *M*, the frequency of oscillation of a vibrating mirror in an interferometric detection setup. This so-called pseudoheterodyne detection^37^ isolates the component of the backscattered radiation that arises from the near-field portion of the optical radiation, and allows the scattered amplitude s_*n*_ and phase φ_*n*_ (at harmonics *n*) to be measured independently. The scattered amplitude and phase relate to the illumination wavelength in a way that is similar to the wavelength dependence of the real and imaginary parts of the dielectric function of the sample, respectively.^38^ For weak oscillators, such as the vibrational modes of the biomolecules probed here, the imaginary part of the dielectric function exhibits peaks at the resonant frequencies of the oscillators.^38^ Thus, the amplitude is suitable for imaging the AuNPs embedded in the cells, whilst the phase is suitable for imaging the cells in a chemically sensitive way. Here we have used s_3_ and φ_3_ (*i.e.* measurements at the 3^rd^ harmonic of the tip frequency) due to their compromise between high signal-to-noise level and low background. The tuneable narrowband QCL light (wavelengths 5–11 μm) allows multiple vibrational modes to be probed and can be tuned to the chemical moieties of interest in biological samples.

## Results and discussion

### Multi-wavelength imaging of hippocampal neurons

First we image neuronal ultrastructure with s-SNOM and we correlate the morphological features with those seen in TEM. As a model system, we chose cultured hippocampal neurons treated with gold nanoparticles (AuNP), because their morphology has been well characterized using both light microscopy and TEM (ref. 39, 40 and ref. therein). To our knowledge, these are the first nanoscale neuronal ultrastructure optical imaging experiments that do not use fluorescent labelling. Dissociated neurons were prepared as previously described^41–43^ and seeded onto coated vinyl coverslips, before being grown for 7 days, fixed, osmicated, dried and processed for TEM.^35^ To provide samples that were suitable for imaging both with s-SNOM and TEM, the samples were sectioned to 70 nm, with the s-SNOM samples mounted on highly reflective silicon substrates to amplify the s-SNOM signal.^44^

**Fig. 2:**
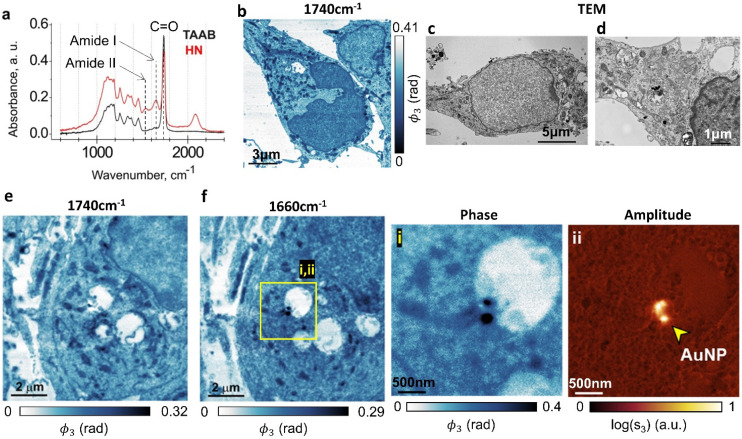
Comparative s-SNOM and TEM imaging of resin-embedded osmicated hippocampal neurons. (a) FTIR absorption spectra of hippocampal neurons embedded in the TAAB 812 resin (HN) and of the embedding resin in cell-free regions (TAAB). Each spectrum is an average of 5 non-overlapping scans. (b–f) s-SNOM (b, e and f) and TEM (c and d) images of osmicated hippocampal neuron sections. s-SNOM images were acquired at 1740 cm^−1^ (b and e) and 1660 cm^−1^ (f). Insets (i) and (ii) show AuNPs internalised in a neuron in s-SNOM phase and amplitude images respectively.

We used a Fourier transform infrared (FTIR) spectrometer to inform our choice of s-SNOM imaging wavelengths. Large-area absorption spectra of the resin-embedded hippocampal neurons and of the adjacent regions that contained only the TAAB resin (Fig. 2a, HN, TAAB) were obtained from far-field FTIR measurements. The resin-embedded neurons showed two characteristic absorption peaks in the 1500–1700 cm^−1^ range originating from the amide moieties that are present in both proteins and nucleobases. The 1650 cm^−1^ amide I band arises both from a C=O stretch mode of amide groups and from C_*x*_=O_*x*_ stretches of guanine/thymine/uracil. The 1540 cm^−1^ amide II absorption band corresponds largely to an N–H bending/C–N stretching mode. The peaks at 1200–1300 cm^−1^ correspond to absorption in DNA/RNA. The antisymmetric phosphate (PO_2_^−^) stretch mode is at 1215/1225/1240 cm^−1^ for Z/B/A-forms respectively,^45,46^ whilst the symmetric phosphate stretch modes, at approximately 1090 cm^−1^,^45,46^ were much less pronounced. The main active vibrational modes excited in these samples are summarised in Table 1.

**Table 1:** Summary of active vibronic modes (non-exhaustive)

Wavenumber (cm^−1^)	Source	Vibronic modes
1738	Embedding resin	C=O stretch
1650	Amide groups	C=O stretch
1540	Amide groups	C–N stretch, N–H bend
1090, 1200–1300	PO_2_ groups	PO_2_ stretch

The FTIR spectrometer averages over a large sample area with a high resin content that dominates the absorption spectrum at these wavelengths, but this does not stop us from using s-SNOM in this wavelength range^29^ for small-area nanoscale chemical mapping. These data agreed with previously reported FTIR spectra of non-embedded hippocampal neurons (900–1800 cm^−1^).^47^

We also note the absorption peak at approximately 2100 cm^−1^ that is exhibited in the neuron sample only. Unfortunately this peak lies outside of the range of our QCL, but it might be a useful spectral feature to probe in future work, should this region of the spectrum be accessible.

Both the absorption spectra have a sharp peak at ∼1738 cm^−1^ (Fig. 2a), largely originating in C=O stretching vibrations of the embedding resin. In the sample region containing neurons, this absorption feature also has contributions from membrane lipids,^48^ the osmium tetroxide (OsO_4_) stain,^49,50^ aldehyde groups in fixatives^51^ and succinic anhydride in resin hardeners.^27^

Taken together, these findings argue that s-SNOM imaging using the amide I absorption band will allow us to locally map the protein/nucleobase distribution across the cellular organelles.^27,29^ They also suggest that imaging at the 1738 cm^−1^ absorption feature should generate images that are most closely correlated with TEM imaging. This is partly because it is imaging the same OsO_4_ stain that generates the TEM contrast, but it is also because at this wavelength, we are measuring the distribution of the resin, and this has a strong negative correlation with the local density of the biomaterial.^52^

Fig. 2 shows s-SNOM images that represent the first direct optical nanoscale images of intracellular structure in neurons. AuNP-treated hippocampal neurons are imaged at two wavelengths. The 1740 cm^−1^ phase images (Fig. 2b and e) show plasma membranes, nuclei, mitochondria and rough endoplasmic reticulum (ER), each identifiable from their morphological correlations with corresponding TEM images of sections prepared in the same way (Fig. 2c and d) which similarly show cell membranes, neuronal nuclei, mitochondria and ER.

The 1660 cm^−1^ image (Fig. 2f) maps the amide density in the cell, with the higher amide content components, be it due to the presence of proteins or of nucleobases, appearing darker in the false colour image. A protein/DNA rich structure, most likely representing nuclear lamina, appears in the nucleus. High amide density mitochondria in the cytoplasm are identifiable by their morphology. Fig. 2e is an s-SNOM image of the same area as Fig. 2f but taken at 1740 cm^−1^, where the nuclear membrane and organelles in the cytoplasm are much more clearly identifiable. We surmise that 1660 cm^−1^ images should be used for mapping amide density in cells, whereas 1740 cm^−1^ is better for detailed studies of membranous structures.

Fig. 2f insets (i & ii) show the s-SNOM phase (i) and amplitude (ii) images for an AuNP-containing region of the neuron; gold has a high reflectivity at this wavelength, making the AuNPs easy to identify in this imaging mode. The phase image (acquired simultaneously) also allows us to map the amide chemical moieties. The combination of the two images therefore enables simultaneous label-free mapping of nanoparticles and cell chemistry in a way that could be used to investigate biochemical changes to the health and function of cellular organelles in response to nanomedicine therapeutics.

### Spatial resolution available for the s-SNOM imaging of neuronal ultrastructure

The spatial resolution in the s-SNOM images depends in a complex way on a large number of parameters (scanning speed, sample morphology, tapping amplitude, tip radius/condition, choice of tip-frequency imaging harmonic *etc*.) and there is always a trade-off between resolution, noise and image acquisition time. A full optimisation of the s-SNOM resolution lies beyond the scope of this paper, but nevertheless, it is instructive to look briefly at the sort of intracellular detail s-SNOM can already provide in these non-optimised and label-free images.

**Fig. 3:**
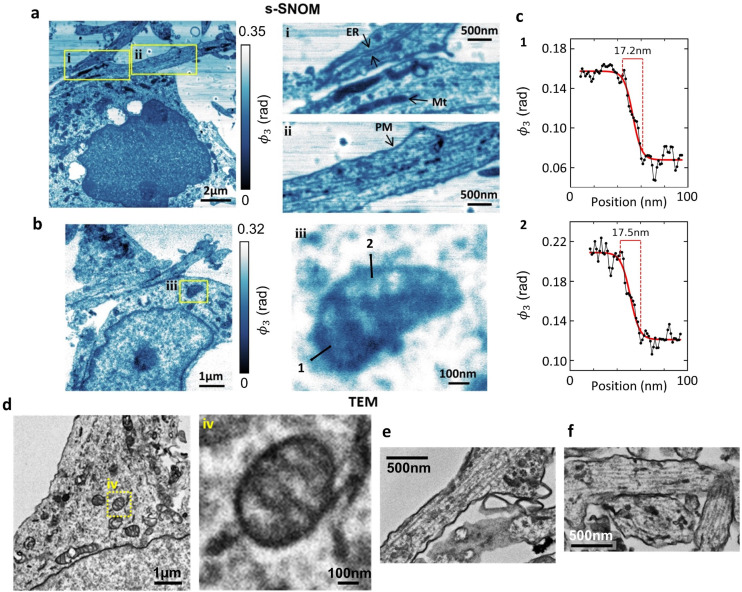
Comparative s-SNOM and TEM imaging of neuronal ultrastructure. s-SNOM images (a and b) of osmicated cultured hippocampal neuron sections acquired at 1740 cm^−1^. Insets, (i) endoplasmic reticulum (ER) and mitochondria (Mt), (ii) the plasma membrane (PM) and (iii) mitochondrial ultrastructure. (c) Spatial profiles of the s-SNOM signal along the lines shown in (iii). The values shown are the 10–90% rise distances of the sigmoid fits (solid lines) to the data. (d–f) TEM images of osmicated hippocampal neurons showing mitochondrial ultrastructure with cristae (d, inset iv) and rough endoplasmic reticulum (e and f).

Using a shorter (2–3 nm) imaging step size (Fig. 3a and b), at the 1740 cm^−1^ wavelength that seems best suited for membrane studies, we can readily resolve (and correlate with TEM) endoplasmic reticulum and plasma membrane (Fig. 3ai & ii, respectively). Several mitochondria are also identifiable (Fig. 3ai & ii), and the mitochondrial ultrastructure (cristae) are seen in Fig. 3biii. These features correlate with their TEM counterparts from the same culture, (Fig. 3d–f) showing mitochondria with clearly defined cristae (Fig. 3div) and a similarly structured ER in neuronal processes (Fig. 3e and f).

Fig. 3c shows spatial profiles of the s-SNOM signal across mitochondrial membranes (lines 1 and 2 in Fig. 3biii), constructed by averaging over a width of 2 pixels (equivalent to 2.6 nm) to reduce noise. The solid curves are sigmoidal fits, and should only be regarded as guides to the eye, but they correspond to 10–90% rise distance (RD_10–90_) values of 17–18 nm.

At the moment the s-SNOM resolution is likely limited by the radius of the tip of the probe,^24^ but we expect that a study aimed at optimising the resolution, possibly using sharper tips, may reduce it towards the ∼1 nm level reported in solid state samples.^53^

Overall, these findings signal the potential for new applications of s-SNOM as a tool to visualize membrane-related cellular processes such as endocytosis, lipid bilayer disruption, nuclear lamina dynamics or drug internalization. This could be in a chemically sensitive way, either alone or in tandem with other complementary imaging techniques.

### s-SNOM imaging of the interaction between nanoparticles and the neuronal ultrastructure

As seen in (Fig. 2fi & ii), the AuNPs are readily detectable with s-SNOM, allowing us to use it as a tool to characterize their interactions with cells.

**Fig. 4:**
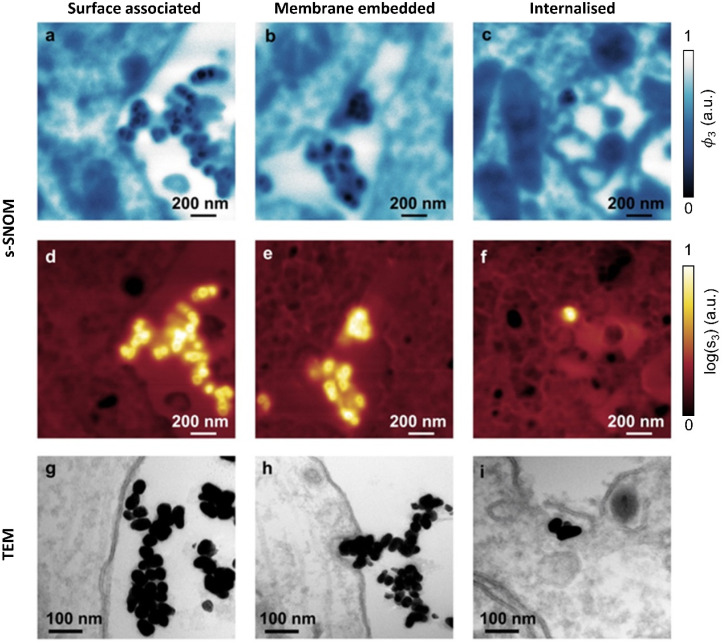
AuNP interaction with hippocampal neurons. Three modes of AuNP interaction with neurons imaged by s-SNOM (a–f) and TEM (g–i). For s-SNOM, both phase (a–c) and amplitude (d–f) images are shown, acquired at 1740 cm^−1^.

Previous TEM studies have shown AuNPs interacting with neurons in several modes. These include surface association (no changes in membrane curvature), membrane embedding (invaginations observed) or internalization.^35^ The 1740 cm^−1^ s-SNOM phase images (Fig. 4a–c) clearly reveal both the AuNPs, (which show up as being darker than the cellular ultrastructure sections and embedding resin) and, simultaneously, the cellular ultrastructure. The s-SNOM amplitude images (Fig. 4d–f) provide confirmation of the AuNP identification.

Both the s-SNOM and TEM images show similar patterns of AuNP distribution. Both show AuNPs in contact with the cell surface (surface associated, Fig. 4a, d and g). Both are also able to detect changes in membrane curvature and embedded AuNP (Fig. 4b, e and h), as well as AuNP internalization (Fig. 4c, f and i). In all cases, s-SNOM phase images were optimal for tracing cell morphology and ultrastructure, whereas amplitude images provided the highest contrast for AuNP detection and localization.

## Conclusions

In summary, we have demonstrated that s-SNOM can be used to map the interactions between nanoparticles and subcellular neuronal structures in a way that is chemically sensitive. We can map functional chemistry, such as amide moieties in organelles that include the nuclear membrane, mitochondria and ER. Furthermore, we find excellent correlation between morphologies seen in s-SNOM and TEM.

To our knowledge, this is the first time that these nanoscale intracellular structures in neurons have been directly and optically imaged.

We have demonstrated that s-SNOM can be used to accurately measure the thickness of the nuclear membrane, and AuNP diameters in a way that agrees with established TEM data. This level of spatial resolution might be further improved by decreasing the tip radius, with as low as ∼5–10 nm (ref. 54) being recently demonstrated with bespoke tips.

These results also suggest a wealth of future applications for s-SNOM in studying unlabelled metal and non-metal nanomaterials in tissues. These could include nanostructures currently being developed for nanomedicine, such as liposomes (IR absorption peaks at 1740 and 1066 cm^−1^),^55^ PLGA (1460, 1750 and 3000 cm^−1^),^56,57^ or chitosan (1420 cm^−1^)^57^ particles. s-SNOM is also expected to be a valuable tool to image drug-loaded nanocarriers by specifically targeting their drug cargo, such as peptides.^58^

## Methods

### Materials

Tetraethylorthosilicate (TEOS), cetyltrimethylammonium bromide (CTAB), sodium hydroxide (NaOH), chloroauric acid (HAuCl_4_), silver nitrate (AgNO_3_), hydrochloric acid (HCl) and sodium citrate dibasic trihydrate, gold chloride trihydrate (HAuCl_4_·3H_2_O), sodium citrate tribasic dehydrate, silver nitrate (AgNO_3_), l-ascorbic acid (AA), hydrogen peroxide solution (H_2_O_2_, 30 wt%), (3-mercaptopropyl), trimethoxysilane (MPTMS, 95%), phosphate buffered saline (PBS, pH 7.4), hydrochloric acid (HCl, 37%), sulphuric acid (H_2_SO_4_, 96%), acetone and 2-propanol were obtained from VWR International, UK. Deionized (DI) water was purified using the Millipore Milli-Q gradient system (>18.2 MΩ).

### Nanoparticle synthesis and characterization

Gold nanospheres (AuNP, average diameter 42 ± 7 nm, mean ± SD) were synthesized using a surfactant free seed-mediated method^59^ as reported in our previous work,^35^ and stored at 4 °C light protected.

To confirm size and morphology of AuNP, the samples were imaged on 300 or 400 mesh copper grids coated with carbon film using bright-field TEM and analysed as we have previously described.^35^

### Cultures of hippocampal neurons

For neuronal cultures, pregnant Wistar rats (E18) were from Charles River (UK). Animals were handled in accordance with European Union (European Directive 2010/63/EU) and UK legislation. Dissociated cultures of hippocampal neurons were prepared and cultured and treated with AuNP as described in our previous articles.^35,41,60^

### Far-field absorbance spectra (FTIR)

Far-field absorbance spectra of neurons were obtained using a commercial Fourier transform infrared (FTIR) spectrometer (Vertex 70, Bruker, USA) with HYPERION IR microscope attachment. For analysis of the hippocampal neurons, TAAB 812 resin-embedded cell sections (1 μm thickness) were deposited onto gold-coated coverslips and absorption spectra were obtained in reflection mode.

### Transmission electron microscopy (TEM)

To study the interaction between AuNP and neurons, dissociated hippocampal neurons (7 DIV) grown on polylysine-coated Thermanox coverslips (Thermo Fisher UK), were treated with AuNP for 24 h, fixed, osmicated, dried, embedded in Taab 812 resin (Sigma-Aldrich, UK) and sectioned to 70 nm as described in our previous article.^35^ Sections were immediately collected on 2 × 1 mm slot grids formvar and carbon coated TEM copper grids (Agar Scientific, UK), dried and stored until TEM analysis. TEM imaging of hippocampal neurons was done at 120 kV (JEOL JEM 1400Plus, JEOL, Japan) with a 2k × 2k format CCD camera (Ruby CCD Camera, JEOL, Japan) as previously described.^35^

### s-SNOM and quantification of signal profiles

s-SNOM images were acquired using a commercial near-field microscope system (neaSNOM, NeaSpec, Germany) and a quantum cascade laser system (MIRcat-QT, Daylight Solutions, USA) with four lasing chips covering a spectral range of ∼(900–1900) cm^−1^. Commercially available probes (Arrow NCPt, NanoWorld, Switzerland) with a resonance frequency *Ω* ∼285 kHz were used and driven at a vertical tapping amplitude of ∼50 nm. A pseudoheterodyne detection setup^37^ was used to demodulate the detected signal, allowing the amplitude and phase of the signal to be measured simultaneously. The signal was demodulated at the third harmonic of the tip oscillation frequency to sufficiently remove the large background in the scattered light from the probe. Image processing such as line noise correction was performed using Gwyddion (v. 2.55) software. To prepare s-SNOM samples, TEM sections of cultured hippocampal neurons obtained as described above (transmission electron microscopy) were collected on silicon wafer chips (Nanoworld), dried and stored until further use.

The 10–90% intensity rise distance (RD_10–90_) method using the sharpness of the edge as an indicator of resolution was applied as previously described.^61^ To calculate RD_10–90_, the upper (100%) and lower (0%) boundaries of the profiles were determined by fitting the profiles to a sigmoid curve, the 10% and 90% levels were calculated, and the data points closest to these values were extracted.

## Author contributions

The project was conceived and co-ordinated by A. E. P. and C. C. P. Hippocampal neurons were embedded and cut into TEM sections by C. M, P. M., and L. A. AuNP were fabricated by C. M. s-SNOM images were acquired and analysed by G. E. G. TEM images of neurons were acquired by P. M. FTIR measurements were performed by G. E. G. The manuscript was written by G. E. G. and C. C. P. with contributions from other authors. All authors have read and acknowledged the manuscript.

## Conflicts of interest

There are no conflicts to declare.

## Supplementary Material
